# Investigation of the Relationship between Genetic and Breeding Characteristics of WBPH Behavior according to Resistant Materials in Rice

**DOI:** 10.3390/plants12152821

**Published:** 2023-07-30

**Authors:** Jae-Ryoung Park, Eun-Gyeong Kim, Yoon-Hee Jang, Sang Yong Nam, Kyung-Min Kim

**Affiliations:** 1 Crop Breeding Division, National Institute of Crop Science, Rural Development Administration, Wanju 55365, Republic of Korea; icd0192@korea.kr; 2 Coastal Agriculture Research Institute, Kyungpook National University, Daegu 41566, Republic of Korea; egk@knu.ac.kr (E.-G.K.); uni@knu.ac.kr (Y.-H.J.); 3 Department of Environmental Horticulture, Graduate School of Sahmyook University, Seoul 01795, Republic of Korea; 4 Natural Science Research Institute, Sahmyook University, Seoul 01795, Republic of Korea; 5 Department of Applied Biosciences, Kyungpook National University, Daegu 41566, Republic of Korea

**Keywords:** plant extract, whitebacked planthopper, rice, environment, behavior

## Abstract

Rice accounts for most of the calories consumed by the world’s population. However, the whitebacked planthopper (WBPH), *Sogatella furcifera* (Horvath), is an insect that can cause rice yield loss. WBPH sucks the stems of rice and negatively affects yield and grain quality. Therefore, numerous insecticides have been developed to control WBPH in rice fields. However, chemical pesticides cause serious problems such as environmental pollution and ecosystem disturbance. Here, we research the possibility of using previously reported rice extracts obtained using methanol, Chrysoeriol 7(C7) and Cochlioquinone-9 (cq-9), as potential insect repellents. WBPH was caged with C7 or cq-9 and monitored, and the WBPH behavior was recorded. The number of WBPHs approaching the periphery of the C7 and cq-9 was very low. In cages containing the C7 and cq-9, only 13 and 7 WBPHs out of 100, respectively, walked around the material. In addition, foliar spraying with C7 and cq-9 did not negatively affect the plant height. The expression level of genes related to resistance was maintained at a high level in the resistant lines when treated with WBPHs alone, but was at a similar level to those of the controls when treated with C7 or cq-9. Interfering with WBPH access did not adversely affect the plant phenotype. Recently, people’s interest in the environment has increased, and the use of plant-derived materials is also increasing. There is a new trend towards using plant extracts as an environmentally friendly means of managing resistance to WBPH during the rice cultivation period, while also avoiding environmental pollution.

## 1. Introduction

Most plants cannot escape from various stresses because they are in a fixed state in which they cannot move on their own. Therefore, in order to endure and overcome this, they have evolved a complex defense system and built a system to protect themselves [1,2,3]. In addition, the defense system to resist stress does not work in a single form, but various factors work in combination, so it cannot be concluded that a change in a plant’s metabolic process is related to a specific stress [4]. In each situation of stress, plants construct various signal pathways, and each of these pathways constantly interacts to help plants overcome the environmental stress [5]. Plants emit aromatic substances or build structural defenses such as thorns to prevent invasion by insects and pathogens [6,7]. In addition, a complex and sophisticated signal transduction system is regulated so that other cells can quickly detect the entry of pathogens and build a defense system [8,9].

Plants synthesize primary metabolites that are directly necessary for growth and development and are highly conserved, and secondary metabolites that are not essential but can defend them against external stimuli or attack by pathogens [10]. In particular, secondary metabolites such as phenolic acids, flavonoids, polyphenols, and stilbenoids are the most common and widely known groups that play a key role in plant resistance responses [11]. Flavonoids act as plant defense substances by inhibiting the growth of pathogens and acting as toxins to them [12]. For example, naringenin is used as a plant defense substance that changes the behavioral characteristics of aphids [13], and flavonoids improve plant defense resistance to symptoms such as the neuroderangement of pests [14].

In recent years, rapid climate change has created many unpredictable situations during the rice growing season [15]. We must always be prepared for unpredictable situations in order to maintain high yield [16]. Whitebacked planthopper (WBPH), *Sogatella furcifera* (*Horvath*), is one of the very important agricultural pests that directly or indirectly affect rice (*Oryza sativa* L.) yield loss [17] and negatively affect yield and grain quality by spreading viruses [18]. WBPH sucks the rice tiller and consumes quantities of plant fluids as a nutrient source. In addition, when a large amount of WBPH occurs in a rice field, a phenomenon called ‘hopperburn’ occurs as the leaves become dry [19]. WBPH is directly involved in rice yield loss and causes additional damage by transmitting grassy stunt viruses and ragged stunt viruses to rice [20]. Ultimately, it causes huge economic losses to farmers [21]. Controlling WBPH in the field includes both inducing changes in WBPH behavior and ultimately killing it. In these circumstances, many farms have relied heavily on chemical pesticides to control WBPH [22,23]. However, when relying on chemical control, unpredictable negative effects such as various environmental pollution and ecosystem destruction occur together [24]. Chemical control also causes acute and chronic poisoning in humans and other pests or animals, as well as in the target pests [25].

In recent years, many people have increased their interest in the environment, and the negative perception of chemical pesticides has become stronger. For this reason, interest in developing pest control agents using plant-derived natural defense substances to replace chemical insecticides has increased [26]. Plant extracts are considered to be biodegradable in the natural environment, have a very low possibility of contamination, and contain very little toxicity [27]. Although some plant extracts have already been reported to have repellent action against pests [28], there is little information available, and research is still in the early stages [29,30]. Plant extracts and natural insecticides that can replace the biological activity and physicochemical properties of chemical insecticides have not so far been developed.

Interest in and demand for eco-friendly agricultural products are increasing worldwide, and the need to reduce the use of organic agriculture and synthetic pesticides has been constantly reported [31]. In addition, it is necessary to develop biological pesticides using plant extracts as an alternative to chemical pesticides that cause many side effects in agricultural environments [32,33]. Research on the potential of secondary metabolites of plants as biological pesticides is being conducted extensively, and biological pesticides will be able to replace synthetic pesticides in the near future [34].

Biological invasions by pests are a major threat to human food, agricultural ecosystems, and crop growth [35]. So far, we have relied on chemical pesticides to solve this problem. However, plant extracts are urgently required to ensure stability in rice harvest levels while reducing the damage suffered during rice cultivation, but without causing problems to the environment, and a lot of research is being conducted on this. C7 and cq-9 were synthesized at high levels in plants resistant to WBPH in previous studies. However, in previous studies, only information on fire quality was studied, and no studies have been conducted on behavioral changes in WBPH. Although these compounds have benzene in their chemical structure, no studies have been conducted on whether benzene causes behavioral changes in WBPH. In this study, in order to analyze the repellent effect on WBPH of rice extracts C7 and cq-9, the behavioral changes of WBPH were investigated using C7 and cq-9 under controlled experimental conditions. All conditions were maintained in the same way except for the C7 and cq-9 treatment. In summary, the rice extracts C7 and cq-9 had repellent effects against WBPH, suggesting that environmentally friendly plant extracts can be used instead of chemical pesticides. Also, this study was conducted to obtain basic data on the possibility of using plant extracts as insect repellents, replacing chemical pesticides.

## 2. Results

### 2.1. Behavioral Changes in WBPH in Response to C7

**Figure 1:**
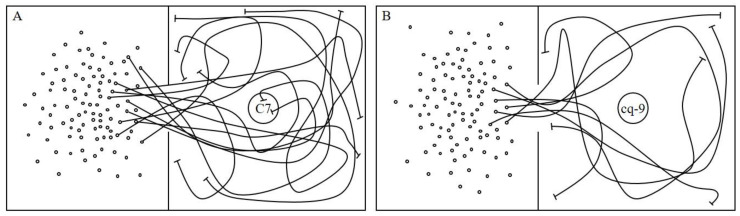
Screening and recording the whitebacked planthopper (WBPH), *Sogatella furcifera* (Horvath), movements for each rice extract. The behavior of 100 WBPH 2-3 instars in cages each containing C7 or cq-9 was investigated. The WBPH 2-3 instars were applied to the avoidance experiment. (**A**) In cages containing C7, 13 of the 100 WBPH 2-3 instars walked around the C7; only two of them sucked the C7 directly. (**B**) Of the 100 WBPH 2-3 instars, only 7 walked around the cq-9; none sucked the cq-9. Hence cq-9 acted more strongly than C7 as a WBPH repellent. The circle indicates the WBPH, the line indicates its movement direction, and the suppression arrow at the end of the line indicates its final position. The circle containing C7 and cq-9 written in the figure means that each plate contains C7 and cq-9.

**Figure 2:**
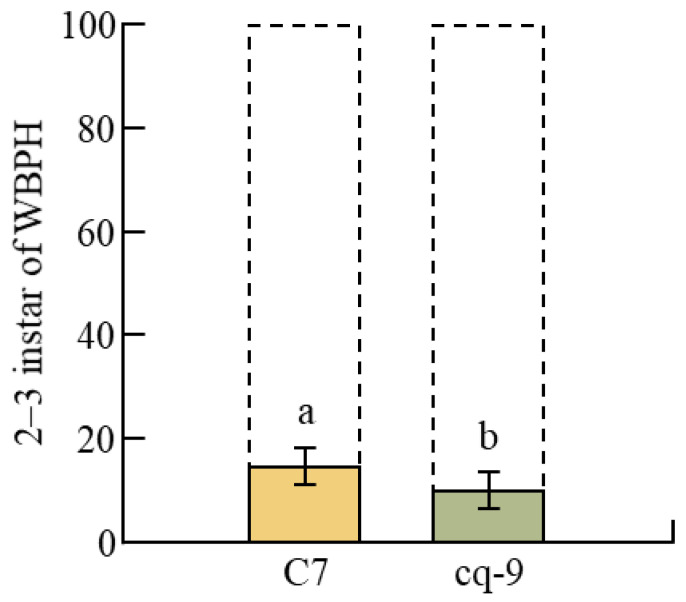
Behavioral comparative analysis of WBPHs for C7 and cq-9. In total, 13 of 100 WBPHs walked around the C7. However, when their behavior was investigated, they passed close to the C7, but then moved as far away from it as possible. So, the last location of the WBPHs is the area close to the wall. Only 2 of 100 WBPHs sucked the C7 directly. Moreover, 7 of 100 WBPHs walked around the cq-9 and there was no direct sucking by WBPHs. Similarly to C7, even in cages with cq-9, the final destination for WBPHs is around the walls. When rice extracts C7 and cq-9 were compared, cq-9 had a greater repellent effect on WBPHs. Bars represent means ± standard error. Means denoted by the same letter are not significantly different (*p* < 0.05), as evaluated by Duncan’s multiple range test (DMRT).

After the extraction of C7, it was placed in a cage with WBPHs. The behavior of 100 WBPHs was monitored for 1 h through a camera, and the path taken by each WBPH were recorded as a line (Figure 1A). An hour-long video was recorded with the camera, and the size of the video is 2.5 GB. The WBPH movements were monitored using images taken from the top to the bottom of the cage, and the movements of each WBPH was marked with a line. A partition wall containing a hole through which the WBPHs could pass was installed in the middle of the cage, and 100 WBPHs were placed in the left part of the partition to monitor behavioral changes. In a cage in which water instead of C7 was placed with the WBPHs, 100 WBPHs actively walked to the left and right sides of the septum in all directions. However, in the cage in which C7 and WBPHs were placed, the behavior was different. Of the 100 WBPHs, only 13 ± 2.3 walked to the right of the septum of the cage (Figure 2). Most of the 13 ± 2.3 WBPHs walked away from the C7. With the exception of only 2 of the 13 ± 2.3 WBPHs, the final destination was near the wall of the cage. In the cage with water, 100 WBPHs displayed the opposite behavior rather than walking along the entire cage.

### 2.2. Behavioral Changes in WBPH in Response to cq-9

The behavioral changes of WBPHs in the case of cq-9 was investigated in the same way as the behavioral changes of WBPHs in the case of C7 (Figure 1B). In a cage containing WBPHs with water instead of cq-9, 100 WBPHs walked to the left and right sides of the bulkhead in all directions and walked throughout the cage. However, in the cage containing cq-9 and WBPHs, only 7 ± 1.2 WBPHs walked to the right side of the bulkhead where the cq-9 was located. Of these 7 ± 1.2 WBPHs, none ingested the cq-9 and all walked around the wall away from the cq-9. Thus, the final destination of the 7 ± 1.2 WBPHs was near the wall, far from the cq-9 (Figure 2).

### 2.3. Changes in C7 and cq-9 Concentration caused by WBPH

**Figure 3:**
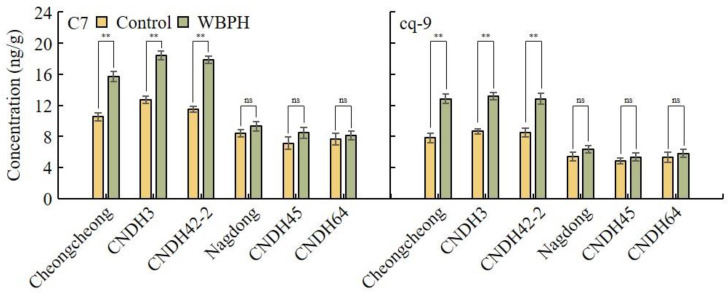
Concentration changes of C7 and cq-9 caused by WBPH. The contents of C7 and cq-9 were changed by WBPH. In particular, the lines resistant to WBPH increased their concentrations of C7 and cq-9 after WBPH treatment. However, in the lines susceptible to WBPH, there was no significant difference in the concentrations of C7 and cq-9, even after WBPH treatment. WBPH-resistant lines: Cheongcheong, CNDH3, CNDH42-2. WBPH-susceptible lines: Nagdong, CNDH45, CNDH64. ^ns^ indicates not significant. ** indicates a significant difference at *p* < 0.01.

The concentrations of C7 and cq-9 were analyzed three weeks after inoculation in the resistant group and in the group susceptible to WBPH (Figure 3). The concentrations of C7 and cq-9 were maintained at higher levels in the control group than in either the susceptible group or in the group resistant to WBPH. Also, when inoculated with WBPH, C7 and cq-9 increased in the susceptible group. However, the degree of increase in C7 and cq-9 was lower than that seen in the resistant group. After WBPH inoculation in Cheongcheong, CNDH3, and CNDH42-2, C7 increased by 49.4%, 44.5%, and 55.0%, and cq-9 increased by 64.5%, 52.4%, and 51.5%, respectively. However, the C7 of Nagdong, CNDH45, and CNDH64 increased by 10.4%, 18.8%, and 5.7%, respectively, and cq-9 increased by 16.6%, 10.1%, and 9.0%, respectively. This suggests that C7 and cq-9 increased to higher levels for resistance enhancement in the WBPH-resistant group.

### 2.4. Changes in Resistance to WBPH Due to Treatment with C7 and cq-9

**Figure 4:**
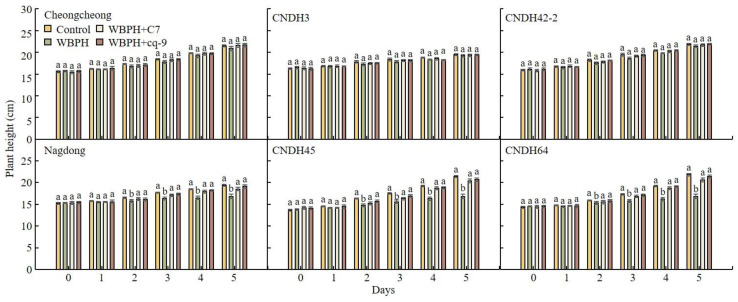
Changes in plant height caused by WBPH and effects of C7 and cq-9. In the lines resistant to WBPH, there was no change in plant height caused by WBPH. However, the plant height after WBPH infection of the lines susceptible to WBPH was smaller than that of the controls. Moreover, when the WBPH-susceptible line was treated with WBPH and C7 or cq-9, the plant height of the controls was the same. No negative effects were observed when plants were sprayed with C7 and cq-9. WBPH-resistant lines: Cheongcheong, CNDH3, CNDH42-2. WBPH-susceptible lines: Nagdong, CNDH45, CNDH64. Bars represent means ± standard error. Means denoted by the same letter are not significantly different (*p* < 0.05), as evaluated by Duncan’s multiple range test (DMRT).

To analyze changes in WBPH resistance due to treatment with C7 and cq-9, plant height was investigated after WBPH inoculation (Figure 4). C7 or cq-9 was foliarly sprayed on each plant, and the plants were then treated with WBPH. The plant heights of Cheongcheong, Nagdong, CNDH3, CNDH42-2, CNDH45, and CNDH64 were investigated on days 0, 1, 2, and 3 after WBPH inoculation, respectively. Cheongcheong, CNDH3, and CNDH42-2 are resistant to WBPH, and there was no significant difference in plant height caused by the WBPH treatment. Moreover, no negative effects on any plant type were observed after treatment with C7 and cq-9. However, Nagdong, CNDH45, and CNDH64 are susceptible to WBPH. When they were inoculated with WBPH, the plants were shorter than the controls, and their growth was negatively affected. However, when Nagdong, CNDH45, and CNDH64 were treated with WBPH and C7 and cq-9, respectively, the plant height was similar to that of the controls. Negative effects on plants caused by C7 and cq-9 were not observed, even in the lines susceptible to WBPH.

### 2.5. WBPH Resistance Score Analysis after Treatment with C7 and cq-9

**Figure 5:**
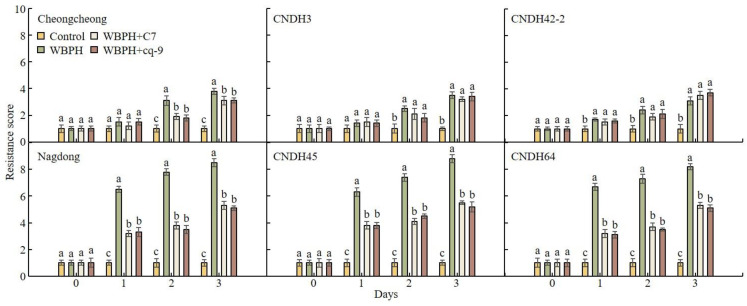
WBPH resistance score analysis. Most of the lines resistant to WBPH had a resistance score of 3 after WBPH inoculation. However, for WBPH, the susceptible lines had a resistance score of 7 after WBPH inoculation. When WBPH-susceptible lines were treated with C7 or cq-9 together with WBPH, the resistance score was lowered by 3 points. WBPH-resistant lines: Cheongcheong, CNDH3, CNDH42-2. WBPH-susceptible lines: Nagdong, CNDH45, CNDH64. Bars represent means ± standard error. Means denoted by the same letter are not significantly different (*p* < 0.05), as evaluated by Duncan’s multiple range test (DMRT).

After WBPH treatment, the resistance score was investigated by assigning RSs to analyze the effect of C7 and cq-9 (Figure 5). Cheongcheong, CNDH3, and CNDH42-2 are groups with resistance to WBPH, and the RSs were all under 5 during the investigation period. In general, an RS level of 5 denotes moderate resistance, so Cheongcheong, CNDH3, and CNDH42-2 showed little damage by WBPH. Also, C7 and cq-9 did not negatively affect any of the plants. However, in Nagdong, CNDH45, and CNDH64, different results were obtained for the different treatments. When Nagdong, CNDH45, and CNDH64 were treated with only WBPH, almost all the plants died, and the RSs were close to 9 after 3 weeks. However, when treated with C7 and cq-9, the RSs were maintained at level 5, which is considered a moderate resistance level. C7 and cq-9 improved resistance to WBPH, but negative effects were not investigated.

### 2.6. Expression Levels of Genes Related to Stress Resistance after Treatment with C7 and cq-9

**Figure 6:**
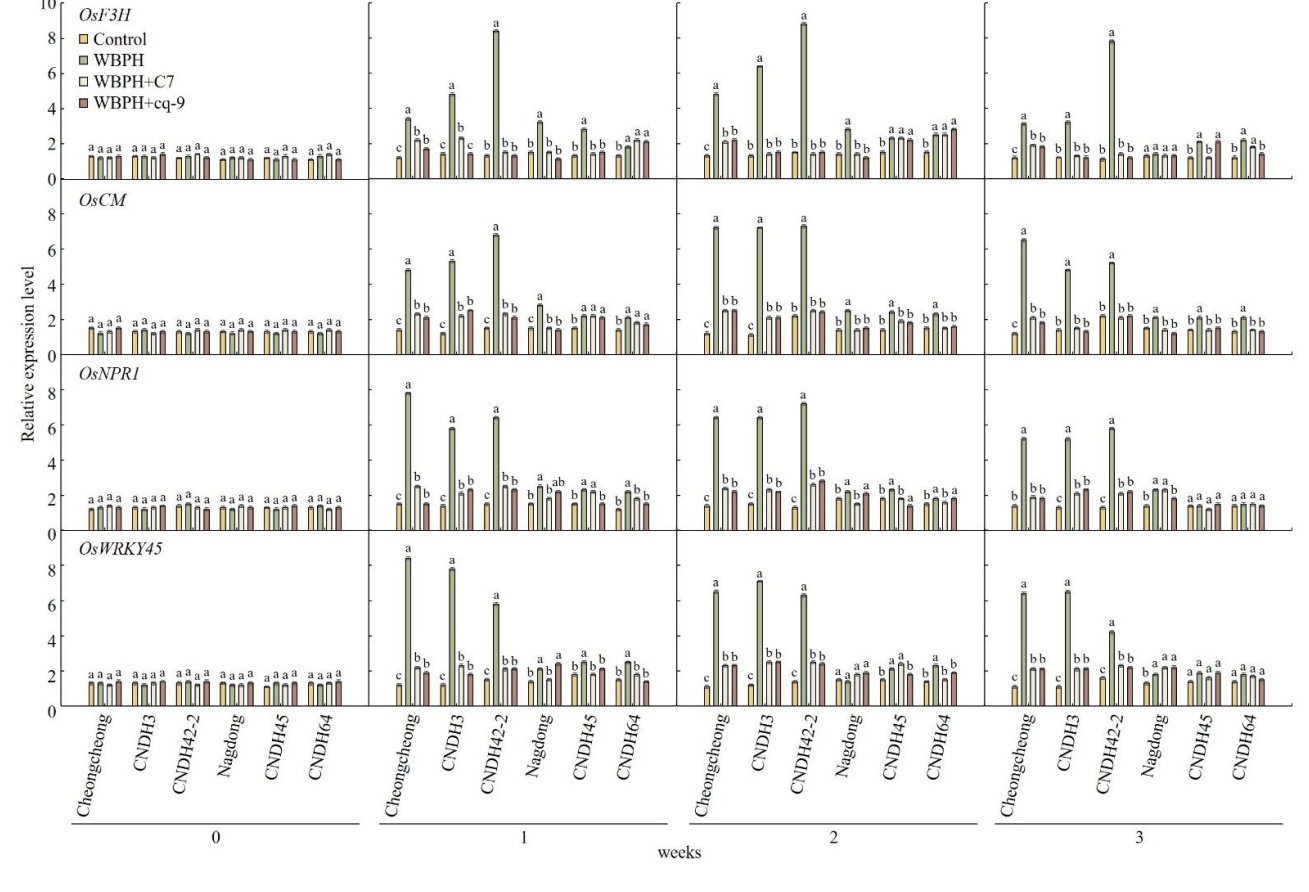
Expression level analysis of plant defense genes after WBPH treatment. In the WBPH-resistant lines, the expression level of plant defense genes increased after WBPH treatment. However, expression did not increase in the WBPH-susceptible lines. In particular, when plants treated with C7 or cq-9 were treated with WBPH, plant defense genes were maintained at levels similar to those of the controls. WBPH-resistant lines: Cheongcheong, CNDH3, CNDH42-2. WBPH-susceptible lines: Nagdong, CNDH45, CNDH64. Bars represent means ± standard error. Means denoted by the same letter are not significantly different (*p* < 0.05), as evaluated by Duncan’s multiple range test (DMRT).

The expression levels of genes related to plant resistance after treatment with C7 and cq-9 were compared and analyzed in Cheongcheong, CNDH3, CNDH42-2, Nagdong, CNDH45, and CNDH64. In Cheongcheong, CNDH3, and CNDH42-2, which are resistant to WBPH, the expression of *OsF3H*, *OsCM*, *OsNPR1*, and *OsWRKY45* was maintained at high levels after inoculation with WBPH (Figure 6). And after treatment with C7 and cq-9, the expression level of the genes used was maintained at a level similar to that of the controls. However, in Nagdong, CNDH45, and CNDH64, which are susceptible to WBPH, the expression of *OsF3H*, *OsCM*, *OsNPR1*, and *OsWRKY45* was maintained at low levels, even after WBPH inoculation. In addition, after treatment with C7 and cq-9, the expression levels of the related genes were maintained at levels similar to those of the controls.

## 3. Discussion

Currently, WBPH is a pest that causes the most significant yield loss in rice [36]. Farmers have implemented chemical controls to minimize the negative impact on grain quality and yield caused by WBPH, but this has a devastating negative impact on the natural ecosystem, including the environment [24]. Recently, the need for natural pesticides or biological pesticides using plant extracts to solve this problem has been continuously noted [37].

C7 and cq-9 are aromatic compounds containing benzene. Plants containing compounds containing benzene establish a defense system as a strategy to hinder the access of insects to their surroundings [38]. In addition, benzene can prepare for stress by sending signals to neighboring plants in addition to defense mechanisms [39]. C7 and cq-9 contain benzene and actually caused changes in the behavior of WBPH. Compared to the controls, the behavior of WBPH in cages with C7 or cq-9 was very static. In addition, foliar spraying with C7 and cq-9 reduced the damage caused by WBPH. Aromatic compounds are precursors of various plant secondary metabolites that play a crucial role in plant growth, development, reproduction, and defense systems, and studies have reported that aromatic compounds are involved in plant defense and signaling using transgenic plants [40]. The chemical formula of C7 is C_16_H_12_O_6_, and it is composed of 5,7-dihydroxy-2-(4-hydroxy-3-methoxyphenyl)chromen-4-one. The molecular weight of C7 is 300.26 g/mol. The chemical formula of cq-9 is C_30_H_44_O_8_, (3R)-9-[(1S,2R,3S)-2-acetyloxy-1,3-dimethylpentyl]-1,2,3,4a,5,6,6a,12 It is composed of 12a,12b-decahydro-12-hydroxy-3-(1-hydroxy-1-methylethyl)-6a,12b-dimethylpyrano 3,2-a]xanthene-8,11-dione.

Secondary metabolites of plants impart various resistances through the composition level of flavonoid compounds, thereby playing an important role in protecting plants from various insects [41]. Secondary metabolites produced by plants can improve the tolerance of plants to abiotic/biotic stress, and are used in various ways such as medicines or food additives [42]. Secondary metabolites act on the interaction between plants and other organisms, including pathogens, and do not play an important role in the growth of plants themselves [43]. Both C7 and cq-9 used in this study are flavonoids [44,45]. C7 and cq-9 are substances related to resistance to WBPH and various other pests [46,47]. C7 is encoded by *OsF3H* [48,49] and cq-9 is encoded by *OsCM*, which regulates their synthesis [50,51]. In leaves previously damaged by WBPH, the content of C7 and cq-9 increased and the expression of various resistance-related transcription factors increased, further strengthening plant resistance [52,53]. This showed the potential of C7 and cq-9 as biopesticides. In addition, C7 and cq-9 are effective not only in controlling WBPH, but also in the control of strains that cause damping off, which is a major disease that affects yield loss by causing major damage at the rice seedling stage [54]. However, in practice, only studies on the degree of resistance and the phenotypic changes of plants when C7 and cq-9 were applied to the leaves were studied, and no study had investigated the behavioral changes of WBPH. C7 and cq-9 are synthesized not only when the plant is attacked by WBPH, but are always synthesized at low levels in rice, and their synthesis is increased under various stressful environmental conditions, including WBPH, and is involved in resistance enhancement [52,53]. Here, when the approach and behavioral changes of WBPH were investigated for C7 and cq-9 extracted from rice leaves, WBPH showed a negative response to the two plant extracts. In the cage containing C7 material, only 2 out of 100 WBPH entered the plate containing the C7 and sucked the juice. However, in the cage containing cq-9, none of the WBPH directly sucked the substance and all just walked away. In addition, when avoidance experiments were conducted on WBPH 4-5 instars, behavioral changes at a level similar to those of 3 instars were investigated. In addition, for WBPH 3 instars and 4-5 instars, the rejection response of WBPH larvae to cq-9 was stronger than the reaction to C7 among the two plant extracts. Here, the number of WBPH nymphs approaching the periphery of the cq-9 was significantly smaller than that of WBPH nymphs recruited around the C7.

When the plants were treated with rice extracts C7 and cq-9, the plant height was not adversely affected. Also, there was no negative effect on bio-scoring. However, what we should focus on is that, when the C7 and cq-9-treated plants were treated with WBPH, there was no significant difference in the expression levels of genes related to resistance improvement. In the WBPH-resistant group, the expression of resistance-related genes was maintained at a high level when treated with WBPH alone. However, when the C7- or cq-9-treated plants were treated with WBPH, the expression level was unchanged at the control level. This means that C7 and cq-9 do not increase the degree of resistance by the plant’s internal attraction. However, it is suggested that they may act to protect plants from WBPH by preventing the access of WBPH to the plant’s surroundings. Summarizing these results, it is reported that C7 and cq-9 may be used as potential environmentally friendly pesticides that can deter WBPH.

Our results showed that the rice-derived substances C7 and cq-9 were involved in repelling WBPH. Biological pesticides using plant extracts have already been studied in various ways [55]. Currently, biopesticides are highly regarded due to their low side effects, high safety, and environmentally friendly properties, and have become a new trend in the development of pesticides for pest control worldwide [55,56]. Biological pesticides developed so far cannot be said to be significantly better than chemical pesticides, but the performance of biological pesticides is also effective. For example, ginkgo biloba extract contains numerous useful components [57]. In particular, flavonoid compounds are being used as insect repellents. The C7 and cq-9 used in this study are also flavonoid compounds involved in the shikimate pathway [58,59]. Flavonoids are typical phenolic compounds and are involved in the growth and development of various pests, viruses, and bacteria by combining with aromatic compounds [60]. In particular, various studies have recently been conducted to replace chemical insecticides with plant extracts [45]. In a study on the insecticidal and repellent effects of chemical insecticides and mosquitoes using plant extracts, the possibility of using plant extracts as alternatives to chemical control was demonstrated [61]. In addition, in terms of the control effect of biological pesticides, rather than expecting a complete replacement of chemical pesticides, the goal is only to reduce the amount of existing chemical pesticides [62].

In order for plant extracts to be considered as possibly suitable for use as pesticides, they should not interfere with plant growth. When C7 was foliarly applied after treatment with brown planthoppers, plant growth was the same or more positive than that without treatment [52]. In addition, when cq-9 was treated with WBPH, plant growth was unchanged or more positively affected [53]. And after the foliar application of C7 and cq-9 to plants, the expression levels of the transcription factors related to plant resistance increased [52,53]. The foliar application of various plant extracts as well as C7 and cq-9 strengthened the resistance system of plants or reduced accessibility by various pests [63,64].

Research on various extracts has increased as interest in plant extracts has increased worldwide as a potential alternative to chemical insecticides [3]. Short-term and long-term field studies are needed to elucidate the applicability of rice-derived C7 and cq-9 as new repellents. In this study, the study was conducted under perfectly controlled conditions. However, in order for C7 and CQ-9 to be practically used by farmers, additional research is needed on how behavioral changes to WBPH occur when treated in the field. Because various environmental factors act on the field, additional research must be conducted to commercially use C7 and cq-9. In order for C7 and cq-9 to be applied as a repellent, a large-scale extraction method and a high-concentration extraction method must be established, and additional research is needed for this purpose. The possibility of using C7 and cq-9 as repellents against WBPH was confirmed in the current in vitro study. We suggest that C7 and cq-9 can be used as stimulants for the development and commercialization of future green pesticides that can replace pesticides based on well-known chemistries.

## 4. Materials and Methods

### 4.1. Rearing of WBPH for Repellent Test

To analyze the behavior of WBPH in response to rice extracts, WBPH distributed by Crop Foundation Division, National Institute of Crop Science was used. The distributed WBPH was reared in a rearing cage (50 cm wide × 50 cm long × 40 cm high) made in the WBPH rearing room in the Plant Molecular Breeding Laboratory of Kyungpook National University. When producing the rearing cage, a transparent acrylic plate was used to allow light to pass through, and a rectangular shape (40 cm wide × 30 cm high) was drilled on three sides to facilitate air flow, and a 100 μm mesh was put in place. A mesh size of 100 μm was able to keep the WBPH out of the rearing cage and facilitate air flow. No holes were drilled in the front. However, a sliding door (40 cm wide × 30 cm high) was made to facilitate the supply of food and water. The WBPH rearing room was maintained at a temperature of 25 ± 1 °C, humidity of 60 ± 5%, light response for 16 h, and dark response for 8 h, and the light intensity was controlled at 3000 lux. As a dietary material for rearing, Chucheong (*Oryza sativa* spp. *japonica* cv. Chucheong), which is susceptible to WBPH, was used. The Chucheong seeds were sterilized with the seed disinfectant Spotak before sowing (Spotak, Hankook Samgong, Seoul, Republic of Korea), and seed treatment agent was used to prevent bakanae disease (Miraebicdyuo, Syngenta, Seoul, Republic of Korea). Each was applied according to the manufacturer’s manual. In addition, seed disinfection and soaking were performed under dark conditions at 33.3 °C for 3 days. The sterilized Chucheong seeds were sown at 5 g each in a plastic container (20 cm wide × 14 cm long × 5 cm high). Hastening of germination was carried out for 3 days under dark conditions at 33.3 °C, and the plant was supplied as a dietary material of WBPH when it reached the 3–4 leaf stage. To subculture the WBPH, generations were advanced by supplying new food every 7 days [65].

### 4.2. Extracting C7 and cq-9 from Rice

Leaves damaged by WBPH were sampled. The sampled leaves were ground into powder form while quenching them with liquid nitrogen using a mortar. Next, 70% methanol was added to make the sample 10 times larger, and was incubated for 16 h at 20 °C and 130 rpm with darkness in a shaking incubator. Thereafter, methanol extract was obtained by filtering three times using filter paper (Whatman Grade 2 Qualitative Filter Paper Diameter: 15.0 cm; pore size: 8 μL). Lipid components and nonpolar impurities were separated using n-hexane in the methanol extract. The filtered methanol extract and the same amount of n-hexane were put together in a separatory funnel and mixed completely. Then, after waiting until the two layers were completely separated, the solution in the lower layer was carefully collected. The collected extract was concentrated using a rotary evaporator. During the concentration, −3 °C cooling water was used and the speed was adjusted in steps of 6–8. Concentration was conducted in a water bath at 30 °C. The Chrysoeriol 7 (C7) and Choclioquinone-9 (cq-9), which are resistant to WBPH, were identified as mobile phases using a TLC silica gel 60F254 plate (Merck, KGaA, Darmstadt, Germany) and a developing solvent in which the ratio of chloroform:methanol:butanol:water was adjusted by 4:5:6:4. Bands corresponding to C7 and cq-9 were scraped from the TLC plate and dissolved in 20 mL of 4% methanol [48,49]. To identify the chemical structure and molecular weight of the extracted material, LC/MS analysis was performed using an MSQ Plus Single Quadrupole Mass Spectrometer (Thermo Fisher Scientific, San Diego, CA Waltham, MA, USA). For analysis, 0.1% formic acid was added to 50% methanol and the extracted sample was diluted at a concentration of 1:1000. The flow rate was set at 50 μL/min.

### 4.3. Evaluation of WBPH Behavior against Rice Extracts

To investigate the repellent effect of the rice extracts on the WBPH, 50 mg of C7 and cq-9 extracted from rice leaves were diluted in 10 mL of 4% methanol and DW in 40 mL of Triton X-100 [0.1 mL/L]. The diluted solution was used for the WBPH avoidance experiment. In addition, cages were specially made to evaluate the potential of the C7 and cq-9 as biopesticides. They were produced using transparent acrylic and measured 50 cm wide × 50 cm long × 40 cm high. A wall was installed in the middle of the cage. At the bottom of this wall, a hole was drilled, through which the WBPH could move. WBPH 3 instars were used to observe the behavior of WBPH in response to the C7 and cq-9. The 3rd instar of WBPH was selected by confirming that the ratio of the fore-wing and the hind wingwas 1:1. In addition, WBPH instars can be distinguished using the number of sensory plates of WBPH. Third instars have 4-5 sensoria [18,66]. A total of 100 WBPH third instars were used, regardless of gender, when examining the avoidance effect of C7 and cq-9 on third instar WBPH. In addition, the WBPH was not supplied with any food other than water for 2 h after collection for use in the test. Based on the middle wall of the cage for testing, it was divided into left and right parts, and WBPH was placed on the left side, and the C7 and cq-9 extracted from rice leaves were placed in each cage on the right side. Then, the behavior of 100 WBPHs in each cage was observed for 20 min. The avoidance experiment for WBPH for each extract was carried out three times independently per extract under controlled environmental conditions (temperature 25 ± 1 °C, humidity 60 ± 5%, light response 16 h, dark response 8 h) in the rearing room. The behavioral changes and lethality of WBPH 3 instars were determined after 20 min. After 20 min, if the WBPH 3 instars did not respond after touching with a brush, they were considered dead. To monitor the WBPH behavioral changes in response to the C7 and cq-9, a camera was installed in the rearing cage and video was recorded. The WBPH behavior was observed for 1 h, and the movements of each WBPH were marked with a line and presented.

### 4.4. Evaluation of Plant Height and Bio-Scoring

Cheongcheong, Nagdong, CNDH3, CNDH42-2, CNDH45, and CNDH64 were selected to evaluate the effects of C7 and cq-9 on rice planthoppers. It has been previously reported that Cheongcheong, CNDH3, and CNDH42-2 are resistant to WBPH, and that Nagdong, CNDH45, and CNDH64 are susceptible [53]. Each seed was disinfected for 24 h using Spotak for disinfection. Afterwards, all the disinfectant solution was removed and the seeds were washed with water and incubated at 33 °C for 3 days in darkness. In order to evaluate resistance to WBPH, 2 rows of seeds for each line were transplanted at an interval of 2.0 × 0.5 cm in a plastic box (32 × 25 × 10 cm), in which 1 row consisted of 12 plants. After sowing, a bioassay for WBPH was performed at the fourth leaf stage. The experiment consisted of 3 independent repetitions. For sufficient inoculation of WBPH, 15 WBPHs were allowed to be concentrated per plant. After WBPH inoculation, resistance scores (RSs) were used to evaluate the response of each line [67]. A resistance score of 9 was assigned when Nagdong, a rice cultivar susceptible to WBPH, died. The RS was investigated based on the change in the phenotype of each plant each day after WBPH inoculation, and the degree of WBPH resistance for each line was evaluated using the average value. After WBPH inoculation, the plant height was investigated in centimeters. Plant height was measured from the base of the stem to the longest leaf.

### 4.5. Leaf Sampling and RNA Extraction

Leaf samples were sampled 0, 1, 2, and 3 days after WBPH inoculation. The sampled leaves were rapidly cooled using liquid nitrogen and stored at −80 °C until use. For RNA extraction, all experimental instruments were washed using RNase inhibitor, and experiments were conducted at 4 °C to increase RNA yield. For RNA extraction, an RNeasy plant mini kit (QIAGEN, Hilden, Germany) was used, and the accompanying manual was followed. In the last step, elution was performed using 50 μL of RNase free water. An ultramicrospectrophotometer ND-2000 (Nanodrop, Waltham, MA, USA) was used to check the concentration and quality of the extracted RNA. In addition, 1 ug of RNA was used for cDNA synthesis. Following the manual provided by the qPCRBIO cDNA Synthesis Kit (PCRBIOSYSTEMS, Cat No. PB30.11-10, PA, USA), cDNA was synthesized to a final volume of 20 μL, and was used as a template for qRT-PCR. qRT-PCR was performed using the Eco Real-Time PCR system. The reaction solution for analyzing the expression level of each gene included 2× qRCRBIO SyGreen Blue Mix 10 μL, cDNA 1 μL, forward primer 0.5 μL (20 pmol/μL), and reverse primer 0.5 μL (20 pmol/μL), and the final volume was adjusted to 20 μL. *OsActin* was used as a control, each reaction was performed 5 times, and the average and standard deviations were calculated.

### 4.6. Statistical Analysis

SPSS software (IMMSPSS Statistics, version 22, IBMSPSS Statistics, version 22, Redmond, WC, USA) was used for the statistical analysis of the investigated values in this study. All experiments were statistically processed using the results obtained from 5 independent experiments. In addition, 10 plants were randomly selected for each study and investigated. A *t*-test was applied to compare and analyze the values of WBPH responses to C7 and cq-9. And for analysis of variance, one-way ANOVA was applied to analyze the survey values. When the *t*-test and one-way ANOVA analysis were performed, when the survey values were analyzed as significant, Duncan’s multiple range test (DMRT) was applied at the significance level of 5%. All experimental results in this study were obtained by independently performing three repetitions.

## 5. Conclusions

Here, the effect of rice extracts on WBPH behavior was investigated. C7 and cq-9 are contained in high levels in WBPH-resistant plants, but their effect on actual WBPH behavior has not been investigated. In this study, the secondary rice metabolites C7 and cq-9 were extracted and WBPH behavioral changes relating to each substance were analyzed under controlled conditions. In this situation, the rice extracts C7 and cq-9 have the potential to replace chemical pesticides. For WBPH, which causes serious damage to rice, avoidance experiments were conducted using C7 and cq-9. In cages containing C7 or cq-9, WBPH barely approached these materials. However, in cages containing water instead of C7 and cq-9, the WBPH roamed freely, regardless of the water. Both C7 and cq-9 had an avoidance effect on WBPH. In particular, the avoidance effect of cq-9 was greater than that of C7. Interestingly, treatment with C7 and cq-9 did not affect plant height: C7 and cq-9 only prevented WBPH access to the plants. In this study, the behavioral changes of WBPH caused by C7 and cq-9 and the effect of the treatment on plants were investigated in a completely controlled environment. However, field tests must be additionally performed in order for C7 and cq-9 to be practically used by farmers because various environmental requirements operate in the field. In the future, various plant extracts require more in-depth study, such as the identification of appropriate concentrations and the use of field tests to understand how extracts may replace chemical pesticides, and to provide basic data for these studies.

## Data Availability

Data will be available upon reasonable request to authors.
